# Fabrication of antibiotic-loaded dissolvable calcium sulfate beads: an in vitro mixing lab utilizing various antibiotic mixing formulas

**DOI:** 10.5194/jbji-6-405-2021

**Published:** 2021-11-10

**Authors:** Edward J. McPherson, Matthew V. Dipane, Madhav Chowdhry, Andrew J. Wassef

**Affiliations:** 1 Department of Orthopaedic Surgery, David Geffen School of Medicine at UCLA, Santa Monica, 90404, USA; 2 Shanti Hospital and Nursing Home, Aligarh, 202001, India; 3 Long Beach Lakewood Orthopedic Institute, Long Beach, 90808, USA

## Abstract

Chronic periprosthetic joint infection (PJI) is a devastating complication that requires an aggressive eradication protocol. Local antimicrobial delivery via dissolvable calcium sulfate (CaSO ${}_{4}$ ) using small-sized beads (3–8 mm) has been utilized as an adjunctive treatment combined with implant exchange, radical debridement, and antimicrobial loaded acrylic spacers. The non-exothermic setting of CaSO ${}_{4}$ theoretically allows for any antimicrobial agent to be used, so long as mixing methods provide a consistent fabrication within a reasonable set time. This study performed the first in vitro mixing study, in which various antimicrobial agents, used singularly and in combination, were mixed with a synthetic CaSO ${}_{4}$ product to observe and document their interactions. The study was performed in a simulated operating room environment. We report a standard mix formula with set times, testing 22 different antimicrobial agents, combinations, and doses. For some antimicrobials and combinations, set times using the standard formula were either too fast or exceedingly slow. For these 14 antimicrobial agents and combinations, we were able to arrive at individualized mixing methods. We present all mixing formulas and set times. In all, we were able to establish mixing methods that incorporate all antimicrobial agents and combinations that we have seen utilized via surgeon-directed use.

## 1 Introduction

Joint replacement surgery for degenerative arthrosis provides improved function and pain relief in the majority of cases where this procedure is utilized (Merx et al., 2003; Lohmander et al., 2006; Lützner et al., 2011; Maradit Kremers et al., 2015). However, adverse responses do occur at relatively low rates (McPherson et al., 2020). Such risks include fixation failure, mechanical dysfunction, fracture, reactive wear debris phenomenon, and infection. Of these complications, that which stands out as the most problematic is periprosthetic joint infection (PJI). This is due to the difficulty in eradicating the infection, as well as the enormous impact placed upon the patient and healthcare system (Kurtz et al., 2008, 2012; Costerton et al., 1999, 2005). Further complicating matters is the extended use of parenteral antimicrobial agents, which disrupts numerous human physiologic systems, confers human biome resistance, and affects future treatment options (Levast et al., 2021).

Alternatives to parenteral antimicrobial treatment for PJI are limited. Local antimicrobial delivery by various carriers has been utilized with increasing frequency in the last 3 decades. The mainstay agents include polymethyl methacrylate (PMMA) in bead form and PMMA in bulk form. In bulk form, the antimicrobial-loaded acrylic cement (ALAC) serves to maintain the joint space. PROSTALAC (PROSThesis + ALAC) implants provide additional stability and function to the resected joint. The other utilized agent for local intra-articular antimicrobial delivery is calcium sulfate (CaSO ${}_{4}$ ), which dissolves and releases antimicrobials. These agents are utilized only for physician-directed use but are considered acceptable methods by those orthopaedic surgeons focusing on PJI treatment (Dacquet et al., 1992; Dahners and Funderburk, 1987; Evrard et al., 1990).

Newer forms of synthetic CaSO ${}_{4}$ have been developed that are considered to be improved products. Legacy CaSO ${}_{4}$ products were “mined and refined” with process techniques that allow implantation into human hosts. Impurities with these products were thought to cause adverse reactions affecting treatment (Lee et al., 2002). Subsequent second- and third-generation products are synthetic, as they are produced from pure elemental form. These agents are better able to accommodate antibiotic impregnation and provide a more consistent performance in terms of mixing, dissolution, and clinical application, although wound drainage still remains a concern for surgeons, with reported rates in the literature varying considerably (Kelly et al., 2001; Ziran et al., 2007; Ferguson et al., 2014; Kallala et al., 2018). Newer antimicrobial delivery agents are being developed for the specific property of antimicrobial compatibility but are not yet in clinical trials (Ambrose et al., 2014). Thus, for the foreseeable future, antimicrobial-loaded CaSO ${}_{4}$ beads, combined with ALAC or PROSTALAC spacers, are the common treatment methods for local antimicrobial delivery.

With the advent of microbial next-generation DNA sequencing combined with specified PJI culture methods, the number of identified microbiota associated with PJI is growing considerably (Aul et al., 1998; Tarabichi et al., 2018). This requires the employment of a wider variety of antimicrobial agents to be used in isolation or in concert. This paper reports our work with a mixing study whereby, in a laboratory setting, we mixed antimicrobial agents into a synthetic, commercially pure CaSO ${}_{4}$ product, creating a dissolvable antimicrobial bead. In cursory review, this concept seems simple, but in reality, the process can be immensely frustrating and difficult. Our purpose is to report our baseline standardized mixing techniques, developing formulas, and doses of antimicrobials added to CaSO ${}_{4}$ . Furthermore, when encountering difficulty in mixing specific antimicrobial agents, we developed modified mixing techniques to create an antimicrobial-loaded bead that previously was problematic with our standard technique.

**Figure 1:**
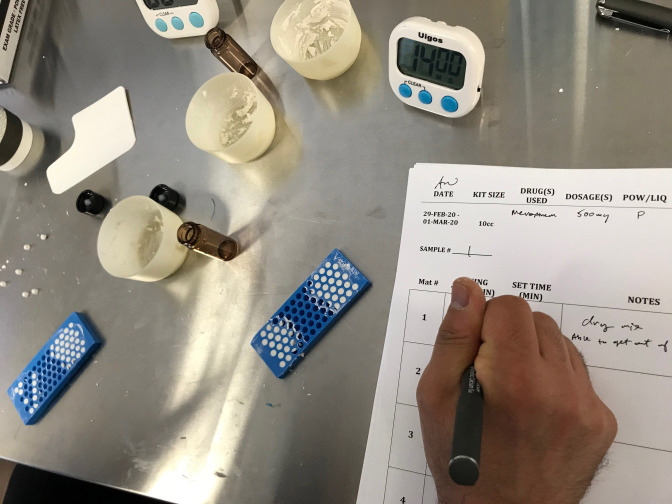
Photograph of 4.8 mm bead mat. The antimicrobial-loaded CaSO ${}_{4}$ paste is shown after spreading the paste into the molds. Once applied, the bead mats were left on the countertop to set, in order to imitate operating room procedure. Time measurements were recorded to establish a set time for each formulation.

## 2 Methods

The CaSO ${}_{4}$ product used for this study was Synthecure^®^ (Austin Medical Ventures, Memphis, TN). We used the 10 cc bead kits that consisted of CaSO ${}_{4}$ hemihydrate powder that is mixed with 5 cc of sterile saline. When mixed, the unloaded CaSO ${}_{4}$ product makes a paste volume of 10 cc. The mixed product is spread into a sterile silicone bead mat, allowing for the fabrication of 3, 4.8, or 6 mm beads. For the purposes of this study, we selected the 4.8 mm beads. The bead mat and bead product formed in this laboratory study are shown in Fig. 1. The antimicrobial formulae selected for study were based on clinical and academic observations of formulae currently utilized in the orthopaedic community.

In the majority of added antimicrobial formulae, the sterile powdered form of the antimicrobial was premixed into the CaSO ${}_{4}$ powder, after which the sterile saline liquid was added. Mixing was performed in a 6 cm diameter mixing bowl with a spatula for 15–45 s, until a paste consistency devoid of antimicrobial clumps was achieved. The paste product was transferred via the spatula to the bead mat. For this study, we measured the time from initial mix until the product became a consistently smooth product devoid of clumps. Once the product was spread onto the bead mat, the product was left to set on the laboratory table in standard conditions of 70 ${}^{\circ }$ F and 40 % humidity. We tested the beads for setting by gently bending the mat at 1 min intervals to assess separation from the silicone mat. In addition, we examined the bead itself. If the bead showed “fissuring” as the mat was gently bent, the curing process was not complete, and the mat was placed back onto the laboratory table. We defined the product as “set” when we could bend the bead mat as it was turned upside down, and the beads would fall onto the table.

A second confirmatory test included a digital bead compression test when the bead was on the table. In some cases, the CaSO ${}_{4}$ product would fall out of the bead mat but was still soft. In this scenario, the beads were tested by compressing the bead with the tip of the index finger. If the bead was not fully cured, the bead would collapse. When “fully set,” the bead would not collapse. Thus, the measured “set time” was defined as the time from initial mix until the bead dropped out of the bead mat and was confirmed as fully set via the digital compression test.

Two exceptions to using the antimicrobial powder mix method for this study were Tobramycin and Gentamicin. In many centers, both Tobramycin and Gentamicin come conveniently premixed in 2 mL vials and can be used as a substitute for the sterile saline that is included in the mix kit. Liquid Tobramycin (or Gentamicin) has two advantages. First, as noted above, it is conveniently provided in 2 mL vials (80 mg of antibiotic per vial). Secondly, as Tobramycin (or Gentamicin) is provided in liquid form, additional antimicrobial powders can be added to the CaSO ${}_{4}$ powder, thus allowing combination formulas to be tested. We elected to use 6 mL of liquid Tobramycin and Gentamicin in our formulae, rather than the manufacturer recommended 5 mL, to make matters simple and efficient for operating room staff, rather than requiring the exact measurement of one-half vial of liquid to achieve 5 mL. Preliminary testing between formulae of 6 mL and those of 5 mL demonstrated no significant difference in set time or quality.

During the standardized mix tests, we found difficulty mixing numerous antimicrobial agents. In many cases, we encountered extremely long set times, ranging from 25 min to over 3 h. Additionally, we experienced antimicrobial–CaSO ${}_{4}$ combinations that set too quickly to effectively mat and fabricate a quality bead product. For these irregular set times, we experimented with alternative mixing methods. We arrived at several mixing techniques to provide reasonable set times. These separate methods are annotated in a separate section where the modified mixing instructions are described in each particular instance. The set time was recorded for each modified technique. The group of modified mixing instructions are grouped into the category of “post-mix protocols.”

For each antimicrobial agent or combination tested, we performed the test a minimum of three times (range three–four tests) between the two authors (Edward J. McPherson, Andrew J. Wassef). The mix time and set time for each antimicrobial agent and combination was then averaged for the reported result.

**Table 1:** Set times of Synthecure with various antimicrobials and combinations.

Antibiotic(s)/antifungal	Dose	Powder (P)/	Avg mix	Avg set	Observations
	(mg)	liquid (L)	time (min:s)	time (min:s)	
Control	n/a	n/a	0:30	4:10	
Amikacin	500	P	0:27	5:35	Easy, smooth mix.
Amphotericin B	250	P	0:52	5:58	Longer to mix into a smooth paste. Mats ok.
Cefazolin	1000	P	0:51	4:41	Thicker paste, doughy. Spreads ok into mat.
Clarithromycin	200	P	0:38	4:30	Smooth mix and mat.
Clindamycin	900	P	0:30	7:03	Slightly runny. Wait 2 min to mat.
Fluconazole	400	P	0:36	4:20	Chalky. Spread into mat quickly.
Gentamicin	240	L	0:25	6:15	Runny. Wait 2 min to mat.
Gentamicin	500	P	0:45	5:48	Easy mix, smooth paste.
Meropenem	500	P	0:43	14:07	Dry mix, comes out of mat at 10 min. Sets on table top.
Rifampin	600	P	0:35	4:23	Need to mix and plate fast.
Tobramycin	240	L	0:22	9:30	Runny. Wait 3 min to mat.
Vancomycin	1000	P	0:40	3:58	Coarse, doughy. Be firm with spatula when matting.
Cefazolin/Gentamicin	1000/240	P/L	0:25	6:57	Easy to mix. Slightly runny. Easy to mat.
Cefazolin/Tobramycin	1000/240	P/L	0:25	9:25	Easy mix.
Clindamycin/Tobramycin	900/240	P/L	0:23	13:32	Runny. Wait 2 min to mat.
Vancomycin/Fluconazole	1000/400	P/P	0:38	4:40	Smooth paste. Easy to mat.
Vancomycin/Gentamicin	1000/240	P/L	0:45	7:44	Runny. Wait 2 min to mat.
Vancomycin/Tobramycin	1000/240	P/L	0:45	7:19	Runny. Wait 2 min to mat.
Clindamycin/Fluconazole/ Tobramycin	900/400/240	P/P/L	0:30	14:30	Sets well. Easy to mix.
Vancomycin/Amphotericin B/ Tobramycin	1000/250/240	P/P/L	0:35	8:13	Runny to start, but do not wait to mat.
Vancomycin/Fluconazole/ Tobramycin	1000/400/240	P/P/L	0:32	8:36	Runny to start. Wait 4 min to mat.
Vancomycin/Rifampin/ Tobramycin	1000/600/240	P/P/L	0:45	10:40	Runny to start. Mild increase in volume. Do not wait to mat.

n/a: not applicable.

## 3 Results

The standard mix times are reported in Table 1. The two measured times include the mix time and set time. In the cases where liquid Tobramycin and Gentamicin were substituted for saline, we also report mix time and set time in a similar fashion. For the standard mix method, all tested antimicrobial(s) were set within 4 to 15 min. The recorded mix times and set times tested between the two testing authors varied no more than 35 s in all agents tested.

**Table 2:** Synthecure set times that required modified mixing techniques.

Antibiotic(s)/antifungal	Dose	Powder (P)/	Avg mix	Avg set	Observations
	(mg)	liquid (L)	time (min:s)	time (min:s)	
Control	n/a	n/a	0:30	4:10	
Ciprofloxacin	1000	P	1:30	7:25	PMP. Add powder at 60 s. Mix for an additional 30 s.
Ciprofloxacin	500	P	1:30	14:30	PMP. Add powder at 60 s. Mix for an additional 30 s.
Cefepime	500	P	1:30	10:20	PMP. Add powder at 60 s. Mix an additional 30 s.
Cefepime	1000	P	1:50	9:10	PMP. Add powder at 60 s. Mix an additional 50 s.
Ceftriaxone	500	P	0:25	19:20	Add an additional 5 cc of saline at beginning of mix.
Daptomycin	500	P	3:00	12:30	PMP. Add powder at 120 s (paste will be almost set). Mix an additional 60 s.
Ertapenem	500	P	2:15	13:15	PMP. Add powder at 120 s (paste will be almost set). Mix an additional 15 s.
Nafcillin	500	P	1:25	12:20	PMP. Add powder at 60 s. Mix an additional 25 s.
Nafcillin	1000	P	1:25	16:30	PMP. Add powder at 60 s. Mix an additional 25 s.
Unasyn	1500	P	2:20	10:10	PMP. Add powder at 120 s (paste will be almost set). Mix an additional 20 s.
Vancomycin/Ciprofloxacin	1000/1000	P/P	1:25	6:30	PMP. Mix Vanc. and CaSO ${}_{4}$ . Wait 60 s before adding Cipro. Mix an additional 25 s.
Vancomycin/Tobramycin	1000/1200	P/P	1:45	17:50	PMP. Mix Vanc. and CaSO ${}_{4}$ . Wait 45 s before adding Tobra. Mix an additional 60 s.
Vancomycin/Zosyn	1000/2250	P/P	2:00	16:15	PMP. Mix Vanc. and CaSO ${}_{4}$ . Wait 90 s before adding Zosyn. Mix an additional 30 s.
Voriconazole/Vancomycin/ Tobramycin	200/1000/240	P/P/L	0:50	22:00	Inject 6 cc of liquid Tobramycin into the vial of Voriconazole. Shake bottle vigorously for 60 s, until all Voriconazole has dissolved. Aspirate 6 cc of liquid from the vial, add to Synthecure + Vancomycin powder, and mix until a smooth paste is achieved.

PMP: post-mix protocol. Mix CaSO ${}_{4}$ with liquid until a smooth paste is achieved. Wait the prescribed time, then add desired antibiotic powder. n/a: not applicable.

The post-mix protocols and described methods are reported in Table 2. For each unique mixing method, we describe the modified mixing technique. The set times were variable, dependent upon the antimicrobial agent(s) added. We will note that we were able to get the CaSO ${}_{4}$ beads to set within a reasonable time with each modified technique. All the recorded mix times and set times between the two testing authors varied no more than 100 s in all agents tested.

## 4 Discussion

Surgeons face a myriad of obstacles in effectively treating PJI. Amongst the most daunting are microbiota reserves hidden within the interstices of bone known as the cortico-canalicular reserve and the retention of “biofilm islands” attached to avascular soft tissue, bone, and residual small pieces of foreign material(s) (Zoller et al., 2020; de Mesy Bentley et al., 2017; Sindeldecker et al., 2020). Parenteral antibiotics have been a traditionally used adjuvant treatment, combined with implant removal, for PJI but have garnered criticism for their harmful effect to host physiology (Levast et al., 2021). Local antimicrobial delivery into the PJI region, on the other hand, is advantageous for many reasons. First, local delivery can provide substantial antimicrobial levels, high enough to diffuse through the biofilm and enact microbiota kill (Brooks et al., 2021; Moore et al., 2021). Similarly, high local concentrations allow for diffusion into the small corticocanalicular systems of bone, thus killing microbiota reserves (Maale, 2009). Even in the event of regional devascularization of bone via debridement, microbiota kill can still occur via antimicrobial diffusion. This is salutary in that suspect bone can be treated with antimicrobial agents. Similarly, the periprosthetic soft tissue envelope partly devascularized by radical debridement can be treated with high levels of antimicrobial agents (Maale et al., 2020). Lastly, local delivery avoids systemic side effects associated with parenteral antibiotics (Levast et al., 2021).

The use of dissolvable antimicrobial-loaded CaSO ${}_{4}$ beads has been utilized for over 3 decades. Its cleared use stems from a process permitting the use of CaSO ${}_{4}$ in the presence of active bone infection dating back to 1997 (Wright Medical Technology, 1997). First-generation products are “mined and refined.” These products are derived from mined gypsum that is subsequently processed to create a medical-grade product that is cleared for insertion in human subjects. Despite their safe use, these products still contain minute amounts of impurities. These minerals include feldspars, dolomite, silica, and corundum (Liu, 2016). These first-generation products have reported variable results and the mineral processing has been thought to be related to side effects, including wound drainage and inflammatory synovitis (Lee et al., 2002). Many companies have since used the above CaSO ${}_{4}$ clearance to create second- and third-generation products that we see today. These products are commercially pure, having undergone manufacturing utilizing pharmaceutical base reagents. The enhanced purity is thought to provide consistency in mixing, dissolution, and application. At present, CaSO ${}_{4}$ is the most common carrier agent that is utilized in local antimicrobial delivery to bone or joint that does not necessitate a second surgical procedure for removal. It is unlikely that new potential carrier systems will enter the market in the near future due to the complicated approval process(es) currently in place.

Our experience in the mixing lab was humbling. First, we encountered antimicrobial–CaSO ${}_{4}$ combinations that set quickly and could not be spread onto the mat fast enough to fabricate quality beads. In cases such as these, we suspect a “hydrophilic steal” phenomenon. Specifically, these antimicrobial powders absorbed H ${}_{2}$ O from the saline additive, depriving the H ${}_{2}$ 0 required to convert calcium sulfate hemihydrate to calcium sulfate dihydrate, which is the final product form. To mitigate fast set times, the antimicrobial powder was added later into the mix, such that the dihydrate was allowed to form. In the extreme case of Ceftriaxone, it was necessary to add additional saline in order to lengthen the set time.

On the other hand, we also encountered prolonged set times, some of which required hours to set with the standard mixing method. In these cases, we suspect “stoichiometric interference.” It is our belief that these antimicrobial powders interfered with the seeding and proliferation of calcium sulfate dihydrate. To mitigate such prolonged set times, we again employed a delayed introduction of the antimicrobial powder to achieve calcium sulfate dihydrate crystal formation. In all instances where we encountered initial difficulties, we were able to create a modified mixing technique to produce a quality bead in a timely fashion.

This compendium of mixing formulas is by no means a recommended treatment algorithm(s) for PJI. Local periprosthetic delivery with antimicrobial-loaded CaSO ${}_{4}$ is being used considerably throughout the world. Up to this point, antibiotic and antifungal formulas used with CaSO ${}_{4}$ in the form of insertable beads have been within the purview of surgeon-to-surgeon or surgeon-to-industry representative sidebar discussions. We wanted to bring these discussions to the forefront. We want to educate those physicians treating bone and joint infections that these antimicrobial formulas exist. Those uninformed in this area of treatment could potentially over-treat with adjuvant parenteral antibiotics, thus causing unwanted side effects. In contrast, studies are slowly emerging that show local delivery via CaSO ${}_{4}$ may mitigate the need for adjuvant parenteral antimicrobial therapy (McPherson et al., 2013). The description of these mixing formulas is to serve as a starting basis for discussion while providing a reference point for set times. We note that it is important to leave the beads undisturbed in the bead mat so that the curing process can take place. Disruption of the curing process could cause incomplete curing, which may lead to more rapid resorption in the body. In the future we hope to establish an open-access site such that all physician and surgeons worldwide can report upon mixing formulas that can help other physicians with difficult infection cases. Additionally, we hope to one day see the expansion of mixing formulas that extend beyond the current realm of antibiotic and antifungal chemicals and transition to other non-specific antimicrobial agents.

## Data Availability

All of our data are presented in Tables 1 and 2.
